# Variegated spatial–temporal landscape of COVID-19 infection in England: findings from spatially filtered multilevel models

**DOI:** 10.1093/pubmed/fdac085

**Published:** 2023-12-21

**Authors:** Wei Zheng, Cecilia Wong

**Affiliations:** Spatial Policy & Analysis Laboratory, Department of Planning and Environmental Management, Manchester Urban Institute, The University of Manchester, Oxford Road, Manchester M13 9PL, UK; Spatial Policy & Analysis Laboratory, Department of Planning and Environmental Management, Manchester Urban Institute, The University of Manchester, Oxford Road, Manchester M13 9PL, UK

**Keywords:** COVID-19 infection, spatially filtered multilevel modelling, spatial-temporal analysis, England

## Abstract

**Background:**

Although there are empirical studies examining COVID-19 infection from a spatial perspective, majority of them focused on the USA and China, and there has been a lacuna of systematic research to unpack the spatial landscape of infection in the UK and its related factors.

**Methods:**

England’s spatial–temporal patterns of COVID-19 infection levels in 2020 were examined via spatial clustering analysis. Spatially filtered multilevel models (SFMLM), capturing both hierarchical and horizontal spatial interactive effects, were applied to identify how different demographic, socio-economic, built environment and spatial contextual variables were associated with varied infection levels over the two waves in 2020.

**Results:**

The fragmented spatial distribution of COVID incidence in the first wave has made a rural–urban shift and resulted in a clearer north–south divide in England throughout 2020. The SFMLM results do not only identify the association between variables at different spatial scales with COVID-19 infection level but also highlight the increasing importance of spatial-dependent effect of the pandemic over time and that the locational spatial contexts also help explain variations in infection rates.

## Introduction

The headline COVID-19 statistics reported by the British government have been at the national or regional level. This lack of systematic spatial–temporal analysis of the footprint of COVID-19 has crippled local responses, leading to political mistrust of government’s local lockdown measures.^1^

Many epidemiologic models have been built and applied under different contexts to predict the COVID-19 incidence and diffusion, such as the susceptible, exposed, infected-infectious and recovered model^2^ and the nonlinear smooth transition autoregressive model.^3^ Social scientists have tried to establish links between COVID-19 incidence with different socio-economic and environmental factors. Most empirical studies have focused on the USA or China, and there has been a lacuna of systematic research to unpack the changing variegated spatial landscape of infections in the UK. The ‘Build Back Fairer: COVID-19 Marmot Review’ highlights that certain spatial settings and environmental conditions are likely to be disproportionately affected by the pandemic and thus may have a link to wider regional inequalities.^1^^,^^4^ Having a rigorous understanding of the spatial landscape of COVID-19 is therefore seen as important to the development of holistic strategies and measures to tackle health and spatial inequalities.^5^

This paper aims to bridge this critical knowledge gap by first exploring the geographic spread and the temporal change and then identify the potential explanatory factors of COVID-19 infection levels in England through spatial clustering analysis and multilevel spatial model methods. Rather than just focusing on various socio-economic and demographic factors, this study examines the often overlooked built environment and locational variables’ relationship with COVID-19 infection. The two key research tasks are: (i) mapping the spatial pattern of COVID-19 infection level and identifying spatial clusters and hot spots to track their changing pattern in 2020 and (ii) examining the changing relationship between different test variables and the COVID-19 infection level over time.

## Research methodology

### Units of analysis and time periods

The core spatial units of analysis are the 6791 Middle Super Output Areas (MSOAs) where COVID-19 test data are officially published and MSOAs (MSOA is the population census geography consisting of 5000–15 000 population each.) were also aggregated to 326 local authority districts (LADs) for spatial modelling. The analytical period involved the 9-month period between 20 March 2020 and 1 January 2021. The changing patterns are examined for two time periods Time Period 1 (TP1) refers to the week ending on 20 March to the week ending on 3 July 2020; and ‘Time Period 2 (TP2)’ is the week ending on 10 July to the week ending on 1 January 2021 (Although the first confirmed case in England was found in late January 2020, local data of infections are only available for public downloading from March 2020.). The rationale of splitting these two time periods is to reflect the decline of COVID-19 cases in summer 2020 to mark the two waves of infection, but the precise cut-off is very much based on the pragmatic reason of capturing consistent official data for spatial comparison. Before 2 July, only the National Health Service swab test results for those with a medical need and critical key workers (Pillar one data: https://coronavirus.data.gov.uk/details/about-data) were released by Public Health England. Since then, the swab tests for the wider population at drive through centres and home testing kits (Pillar two data: https://coronavirus.data.gov.uk/about) were also included for daily publication. We only focus on infection levels in 2020 to control the impact and the interactive effect brought by the vaccination programme since 2021.

### Variables

The key explanatory variable of this study is the COVID-19 infection level by examining the official data of ‘people tested positive per 100 000 population’ for spatial modelling. The lack of pillar 2 data in TP1 means that our understanding of the real gravity of COVID-19 circulation in the first wave would be constrained. It is due to this varied data compilation problem, the location quotient (LQ) of the COVID-19 positive cases was used to map the spatial clusters/outliers via calculating Local Moran’s *I* values. The use of LQ allows us to examine the spatial concentration of COVID levels by benchmarking the proportion of cases in England as a whole. The formula of LQ is


(1) \begin{equation*} \mathrm{LQ}i=\left( Xi/\Sigma Xi\right)/\left( Pi/\Sigma Pi\right)\ast 100 \end{equation*}

in which *X_i_* is the number of infections in MSOA*i* in a particular period (TP1/TP2), Σ*X_i_* is the sum of infection cases of all MSOAs, *P_i_* denotes the population in MSOA*i* and Σ*P_i_* refers to the total population of all MSOAs. The value of 100 signifies the same infection level of England, under 100 shows less infection level than England and over 100 means above English level of infection.

A range of test variables, informed by literature and official reports, covering demographic, socio-economic, built environment and locational dimensions were included at MSOA and/or LAD level (see the supplementary document):

### Spatial analytical methods

#### Spatial clustering analysis

Local patterns of spatial association were identified by mapping Local Moran’s *I* values^6^ of the LQ of COVID-19 infection rate to detect local clusters and outliers. The equation for calculating Local Moran’s *I* statistic is


(2) \begin{equation*} Ii=\frac{\left({x}_i-\bar{x} \right)}{S_i^2}\times \quad{\varSigma}_{j=1}^n{W}_{i,\,j}\left({x}_j-\bar{x} \right), \end{equation*}

where *I_i_* represents Local Moran’s *I* statistic for MSOA*i*; *x_i_* is the LQ value for MSOA*i*; *x_j_* is the LQ for MSOA*i*’s neighbouring MSOA*j* (*i* ≠ *j*); *-* denotes the mean LQ value of all observations; *W_i,j_* is the spatial weight between MSOA*i* and neighbouring areas *j*; and *S_i_* represents the deviation value for *i*. The method has limitations regarding how to decide the spatial contiguity matrix objectively and how not being linked to scaling laws.^7^ Local Moran’s *I* values in this study were calculated via the Mapping Clusters toolset in ArcGIS Pro. Based on distance decay function, the spatial weight between *i* and *j* was determined by the inverse distance setting. The Local Moran’s *I* index together with its computed *z*-score and *P*-value were used to derive four statistically significant spatial groups: (i) high-value cluster (HH); (ii) low-value cluster (LL); (iii) high-to-low value outlier (HL); and (iv) low-to-high value outlier (LH).

#### Spatial modelling approaches

The multilevel model (MLM), eigenvector spatial-filtered single-level model (ESFSM) and spatially filtered MLM (SFMLM) were used to examine the relationship between the test variables and COVID-19 infection rates. The application of the MLM can capture variations in the explanatory variables at the MSOA and LAD levels but not the spatial autocorrelation at each horizontal level. The ESFSM can produce a spatial signal to explain spatial autocorrelation in the residuals and to ensure the independence of the error term.^8^^,^^9^ To capture both vertical and horizontal spatial dependency effects of COVID-19 infection level, the spatial filtering process is introduced to a multilevel setting. The SFMLM is specified as


(3) \begin{align*} &{Y}_{ij}={\alpha}_{00}+{\varSigma}_{m=1}^M{\beta}_m{X}_{mij}+{\varSigma}_{n=1}^N{\gamma}_n{Z}_{nj}+{\delta}_0 \nonumber \\ & \quad +{\delta}_1e1+\dots +{\delta}_n en+{u}_{0j}^{\prime }+{r}_{ij}^{\prime } \end{align*}

in which the spatial filtering component *δ*_0_ *+ δ_1_e*_1_ *+ ^…^ + δ_n_e_n_* could account for the spatial effect defined by the Moran eigenvectors; and the component *u’*_0*j*_ *+ r’_ij_* is the white noise that is independent. The eigenvector filtering process is applied to the MSOA level.

All the model analyses were performed in the R environment by mainly referring to the ‘lme4’,^10^ ‘spdep’^11^ and ‘spmoran’^12^ packages. The Akaike Information Criterion (AIC) was applied to compare model fitness, with a lower value representing better fitness.^11^^,^^13^ Details of the three models could be seen in the supplementary document.

## Results

### Spatial clustering of COVID-19 infection level

**Fig. 1:**
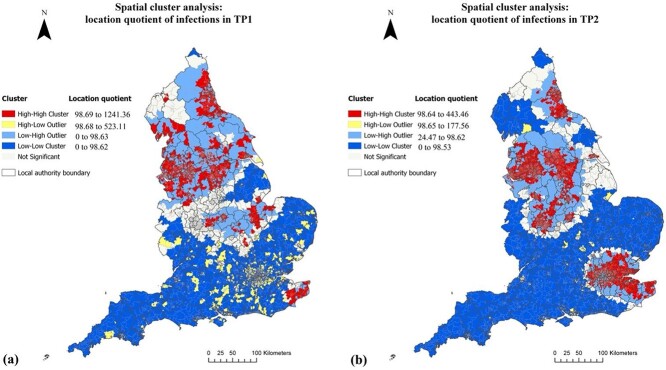
Spatial cluster analysis of LQ of COVID-19 infection rate in TP1 (**a**) and TP2 (**b**). Contains data from OS data and coronavirus data (https://coronavirus.data.gov.uk/) @Crown copyright and database right.

The spatial movement of the hotspots and outliers of COVID-19 infection LQ over the two time periods is shown in Fig. 1. While COVID hotspots (HH cluster) in *TP1* were spreading across different parts of England, they tended to be found in northern England with the M62 motorway corridor as the dividing line. The most affected areas were Merseyside, Greater Manchester, South Yorkshire, Tyne and Wear and Teesside as well as parts of Lancashire and Cumbria. The remaining northern England largely felt into the LH outlier group as the entire area was vulnerable to the spread of the virus. There were a lot of areas falling into the precarious categories and could not be classified, including part of the Midlands. However, some hot spots were scattering in the Midlands with larger clusters found in the Peterborough and Huntingdonshire area and in north of Warwickshire. In southern England, a lot of small outliers with high COVID levels were surrounded by neighbouring areas with much lower infection levels; and London was the main outlying area with much higher infection levels than the adjacent areas. The only COVID hotspot in the south during TP1 was in Kent.

The notable spatial change of infection in TP2 was characterised by a north–south divide along the Severn-Wash line and an urban–rural divide. The rural–urban shift of infection was witnessed between the two waves as most outliers in the shire areas turned into cold spots, which was true in southern England as well as in Cumbria and East Ridings of Yorkshire in the north. The COVID-19 hotspot footprint (HH) in TP2 has become a mirror image of the functional urban areas, as defined by Eurostat’s Urban Audit. These combined effects mean that the northern cities and their hinterland were most affected by the pandemic throughout 2020. However, some hotspots in TP1 have become cold spots (LL) in TP2 (e.g. Peterborough and Huntingdonshire area), whereas most shire areas in the South West remained to be cold spots throughout. Some HL isolated outliers in TP1 have turned to hotspots in TP2 by spreading the virus to its neighbours, with MSOAs in and surrounding Greater London as the notable examples. Kent, nonetheless, continued suffering from being the hotspot, especially with the Alpha variant found there.

### Spatial modelling results of COVID-19 infection level

**Fig. 2:**
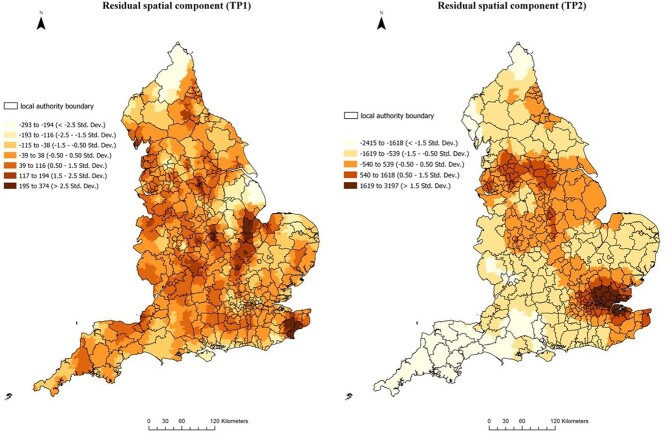
Residual spatial component via eigenvector spatial filtering of SFMLM 1 and SFMLM 2. Contains data from OS data and coronavirus data (https://coronavirus.data.gov.uk/) @Crown copyright and database right.

A comparison of AIC values of the three models for TP1 suggests that the test variables in SFMLM1, together with the multilevel and spatial dependency effects, explain 51% of variance in the COVID-19 infection rate in TP1 and that it has the highest adjusted R^2^ and lowest AIC values (see Table 1). The SFMLM1, therefore, is the best-fit model for TP1. Similarly, the SFMLM2 has the best model fitness among the three models examining the infection rate in TP2 (see Table 2). Figure 2 maps the residual spatial component predicted by the eigenvector spatial filtering process of TP1 and TP2. More modelling fitness details are explained in the supplementary document.

**Table 1:** Estimation results on the Covid-19 infection rate in TP1

Variable	Null model 1	MLM 1	ESFSM 1	SFMLM 1
MSOA level				
% Population aged 16–25	—	−539.31^***^	−558.64^***^	−529.18^***^
% Population aged 70+	—	290.71^***^	262.68^***^	366.95^***^
% Households with 4 people	—	36.93	87.67	43.39
% Usual residents living in a communal establishment	—	787.38^***^	842.86^***^	784.45^***^
% Population—Black	—	15.13	−18.35	36.63
% Population—Indian	—	588.44^***^	812.42^***^	582.64^***^
% Population—Pakistani	—	455.38^***^	313.11^***^	458.96^***^
% Population—Bangladeshi	—	−375.67^***^	−339.18^***^	−304.32^***^
% Population—Chinese	—	−1811.21^***^	−1861.34^***^	−1632.83^***^
% Population—other Asian	—	889.25^***^	320.26	1065.99^***^
% Population working from home	—	−1228.79^***^	−1374.78^***^	−1191.29^***^
% Population working in the health sector	—	393.93^***^	400.24^***^	387.21^***^
IMD rank	—	−0.02^***^	−0.01^***^	−0.02^***^
% Built-up area	—	25.21^**^	9.86	26.77^***^
LAD level				
% in employment as managers, directors and senior officials	—	−38.75	95.72	−42.84
% in employment in associate professional & technical jobs	—	232.10	199.70^**^	51.21
% in employment in sales and customer service jobs	—	293.38	227.02^**^	261.37
% in employment in process, plant and machine operatives	—	323.86	337.31^***^	171.26
% in employment in skilled trades jobs	—	−165.41	233.29^**^	88.22
Affluent England	—	41.84^**^	12.19	31.10^**^
Business, education and heritage centres	—	24.35	5.12	21.40
Countryside living	—	22.00	27.43^***^	17.71
London cosmopolitan	—	−19.05	−26.31	−9.93
Services and industry legacy	—	−27.04	−12.82	−13.64
Urban settlements	—	−5.65	3.61	−0.12
East of England	—	−10.55	36.09^***^	−12.60
North East	—	138.06^***^	259.83^***^	125.63^***^
North West	—	164.46^***^	168.65^***^	142.87^***^
South East	—	−3.30	47.59^***^	2.55
South West	—	−124.25^***^	−23.87	−129.58^***^
West Midlands	—	18.66	−42.84^***^	−26.71
Yorkshire and the Humber	—	86.48^***^	122.75^***^	62.76^**^
Random effects				
Variance at the MSOA level	42 120	34 027	—	—
Variance at the LAD level	21 746	10 001	—	—
Standard error of spatial effects	—	—	74.23	100.62
Moran’s *I* of the estimated spatial process	—	—	0.157	0.056
Intercept	301.33^***^	285.31^***^	231.41^***^	294.20^***^
Adjusted *R*^2^	—	0.478	0.373	0.511
Log likelihood	−46 171	−45 368	−45 659	−45 256
AIC	92 349	90 806	91 404	90 586
BIC	92 369	91 045	91 697	90 839

Note: ^**^p < 0.05; ^***^p < 0.01.

**Table 2:** Estimation results on the Covid-19 infection rate in TP2

Variable	Null model 2	MLM 2	ESFSM 2	SFMLM 2
MSOA level				
% Population aged 16–25	—	5845.19^***^	5574.57^***^	5975.32^***^
% Population aged 70+	—	1326.10^***^	1232.29^***^	1365.99^***^
% Households with four people	—	3151.67^***^	4409.12^***^	2928.71^***^
% Usual residents living in a communal establishment	—	1832.20^***^	1446.11^***^	1880.96^***^
% Population—Black	—	−1032.51^***^	−880.30^***^	−1685.42^***^
% Population—Indian	—	3075.75^***^	3278.22^***^	2788.47^***^
% Population—Pakistani	—	3448.89^***^	3314.66^***^	3358.50^***^
% Population—Bangladeshi	—	857.21	2119.39^***^	1145.76^**^
% Population—Chinese	—	−14 636.86^***^	−10 695.62^***^	−15 591.24^***^
% Population—other Asian	—	−2573.78^***^	−7515.88^***^	−2004.34^**^
% Population working from home	—	−4750.80^***^	−7941.14^***^	−4203.48^***^
% Population working in the health sector	—	311.38^***^	321.77^***^	360.71^***^
IMD rank	—	0.001	−0.03^***^	−0.02
% Built-up area	—	36.29	164.91^***^	−38.32
LAD level				
% in employment as managers, directors and senior officials	—	−2905.78^**^	−4135.78^***^	−2004.35^***^
% in employment in associate professional and technical jobs	—	1172.66	795.33^**^	−54.97
% in employment in sales and customer service jobs	—	−2106.62	−165.84^***^	1304.58
% in employment in process, plant and machine operatives	—	2250.20	1885.3^***^	1636.97
% in employment in skilled trades jobs	—	−4950.58^***^	−3986.98^***^	−1863.47^**^
Affluent England	—	−259.48^***^	−154.09^***^	−185.82^***^
Business, education and heritage centres	—	−180.54^***^	−3.04	−71.94
Countryside living	—	−131.29	−116.14^***^	−22.08
London cosmopolitan	—	79.61	−535.14^**^	−210.51
Services and industry legacy	—	−116.47	112.68^**^	−39.67
Urban settlements	—	−291.73^***^	176.22^***^	−87.10
East of England	—	−402.65^**^	−889.38^***^	−250.31
North East	—	574.65	259.17^***^	896.27^**^
North West	—	762.27^***^	−643.21^***^	952.47^***^
South East	—	−535.52^***^	138.62^**^	337.29^**^
South West	—	−1644.45^***^	−207.65^***^	241.79
West Midlands	—	−479.84^**^	384.65^***^	248.98
Yorkshire and the Humber	—	36.07	−211.97^***^	273.87
Random effects				
Variance at the MSOA level	707 455	484 389	—	—
Variance at the LAD level	17 40 129	882 701	—	—
Standard error of spatial effects	—	—	954.33	202.30
Moran’s *I* of the estimated spatial process	—	—	0.359	0.357
Intercept	3244.58^***^	3151.81^***^	3048.57^***^	2344.57^***^
Adjusted *R*^2^	—	0.795	0.652	0.829
Log likelihood	−55 990	−54 656	−55 977	−54 282
AIC	111 986	109 382	112 031	108 637
BIC	112 006	109 621	112 298	108 889

Note: ^**^p < 0.05; ^***^p < 0.01.

When comparing SFMLM1 and SFMLM2 (see Tables 1 and 2), both the elderly population group (% aged ≥70) and ‘% usual residents living in a communal establishment’ had significant and positive coefficients for both time periods. The younger aged group (% aged 16–25), however, had an inverse relationship with infection rates in TP1 but was found to be strongly and positively related to infection rate in TP2. Household size also manifested a major shift between the two waves, with ‘% households with four people’ not significantly correlated to the infection rate in TP1 but showing a strong positive association in TP2.

Areas with high proportion of Indian and Pakistani population groups were found to be badly affected by the pandemic in both time periods with strong positive coefficients, whereas the situation with the Chinese population group was the opposite. The relationship for other ethnic groups was more mixed. The Black population group, without a significant coefficient in TP1, had a negative coefficient in TP2. The other Asian population group, found to be positively associated with infection rates in TP1, flipped to a negative coefficient in TP2, but the situation was the exact opposite for the Bangladeshi group.

Two socio-economic variables performed the same over TP1 and TP2: ‘% population working from home’ with negative coefficient and ‘% population who work in the health sector’ having positive coefficients. None of the other occupational variables showed any significant relationship with infection level in TP1. The situation changed in TP2, as areas with large proportion of high-end jobs, ‘% people in employment who are managers, directors and senior officials’ and ‘% people in employment who are in skilled trades jobs’ were negatively related to infection level.

Area density, measured by ‘% built-up area’, showed a positive but weak relationship with infection level in TP1 but the effect became insignificant in TP2. The other factor is the IMD rank, which had a significant but very weak negative effect in TP1 but the effect became insignificant in TP2.

Finally, ‘Affluent England’ was found with a small positive relationship with infection level in TP1, though it turned negative in TP2. The ‘North West’ and ‘North East’ were the two regions that consistently had strong positive coefficients in both models. ‘Yorkshire and The Humber’ showed a positive but small effect in TP1 whereas the ‘South East’ (mainly due to the spread of the Kent variant) had a positive coefficient in TP2. The ‘South West’ had a negative coefficient in TP1.

## Discussion

### Main findings

The results from spatial clustering analysis and SFMLM suggest that the original spread of the outbreak was more random. With the advance of the pandemic, the virus tended to spread outwards locally and a more spatially dependent pattern emerged in TP2. The changing spatial dependency effects captured by SFMLMs over time demonstrate the role of geography in disease transmission, particularly during the pandemic.^14^ This also points to the relevance of local lockdown measures implemented in 2020. Thus, a dynamic and multiscalar spatial approach is much needed to develop evidence to track the changing transmission geography to inform policymaking at different levels.

The SFMLM modelling results confirm the importance of wider regional contextual effect: the North West and North East regions are found to be significantly correlated with the infection rate throughout the pandemic. Due to the Kent variant, the South East regional effect was also significant in the second wave, though less strong than the two northern regions. On the opposite end, the South West and Affluent England are found negatively related to infection rate in TP1 and TP2, respectively. The disproportionate concentration of infections in the northern regions is largely related to their demographic and occupational compositions, but it is important to note that extra regional contextual effects are picked up by the SFMLMs. This suggests that there has been failure in controlling the spread of the virus in these regions beyond the test variables, which could be related to the interplay between central-local policy measures and resource inputs to these regions, or behavioural factors that are not captured by the models. Only Affluent England has an inverse relationship with infection level in TP2 but that mainly refers to the affluent South East hinterland of London. This confirms that except the most affluents, other areas are more vulnerable to the infection risk, especially those with a large proportion of disadvantaged groups.

While some explanatory factors became less important between the two waves, others became more influential. There were strict national lockdown measures in TP1, but only national restrictive measures and local lockdown were in place during TP2. This means that the changing explanatory power of different factors was an interactive outcome with changing government measures. One notable change was the local transmission effect in TP2 and hence large household size came into play, so were the younger population groups who returned to schools and universities. Another change was associated with the IMD rank, which suggested more deprived areas tended to observe higher infection levels at the early stage of the pandemic, though this effect has washed away. The wider circulation across schools and colleges could have dampened the deprivation effect and the government’s local measures and other factors might have taken effects too. With national lockdown in TP1, the risk of exposure across different occupation groups was insignificant; however, those in managerial and skilled occupations who are more adaptable to online working had a negative relationship with infection rates in TP2. This highlights the need to have workplace mitigation measures, especially those who require close personal contact.

### What has already been known on this topic?

Commonly reported test variables in relation to the COVID-19 cases are demographic variables, such as population, gender and age structure, with the inclusion of household size in some studies.^15^^,^^16^ Different socio-economic groups have also been reported to possess variated risks of exposure widely.^17^ Some studies go further to explore the wider locational activities such as globalization and GDP levels.^15^^,^^18–20^ Population movement and population density have also been reported to influence the spatio-temporal distribution of COVID-19 cases.^15^^,^^21^^,^^22^ Built environment attributes, such as commercial vitality, transport density and network accessibility, are found directly or indirectly related to COVID-19 spread in different contexts.^23^^,^^24^ However, mixed findings have been uncovered under different contexts.

### What this study adds?

This study provides insights on the spatio-temporal landscape of COVID-19 infection in England. Besides various socio-economic and demographic factors, this study also examines the often-overlooked built environment and locational variables’ relationship with COVID infection. This study adopts an SFMLM approach that captures both hierarchical and horizontal spatial interactive effects to examine how different variables at the MSOA and LAD levels are correlated with COVID-19 infection. This goes beyond the common use of either single-level spatial models or multilevel (non-spatial) models. Most health related variables exhibit spatial dependency, whereas health outcome data are characterised by a hierarchical structure. The application of SFMLM in this study provides useful insights for future studies under different contexts.

### Limitations of this study

Due to the availability test data in TP1, the actual community infection levels over different locations could not be established. The ONS COVID-19 infection survey data (https://www.ons.gov.uk/peoplepopulationandcommunity/healthandsocialcare/conditionsanddiseases/datasets/coronaviruscovid19infectionsurveydata/2020) are designed to establish the global infection level rather than providing accurate geographical distribution data to validate the official data. The lack of consistent COVID-19 test data and the use of 2011 census data for a few test variables have also limited the robustness of the modelling, especially for TP1.

## Conflict of interest

The authors declare that they have no conflicts of interest in this work.

## Supplementary Material

Supplementary_document_clean_fdac085

Supplementary_document_track_changes_fdac085

## Notes

**Wei Zheng,** Leverhulme Early Career Fellow

**Cecilia Wong,** Professor of Spatial Planning
