# Supplementary material for: Variegated spatial–temporal landscape of COVID-19 infection in England: findings from spatially filtered multilevel models
Source: J Public Health (Oxf). 2023 Dec 21;45(Suppl 1):i45–53. doi: 10.1093/pubmed/fdac085 (PMC10734670; doi:10.1093/pubmed/fdac085)
Supplement: Supplementary_document_clean_fdac085 [file supplementary_document_clean_fdac085.docx]

**Technical Annex**

**Variables: definition and rationale**

*Demographic variables:*

- Age: *‘% of population aged 70 and over’* and *‘% of population aged 16 to 25’* were included in the study because people over 70 are consistently identified to be clinically vulnerable^[[1]](#footnote-1)^ and infection cases in Britain since mid-2020 has been related to the younger population.
- Ethnicity: *‘% of population from different ethnic groups’* was included to reflect government reports that COVID-19 has a larger impact on the BAME group.
- Household: *‘% households with 4 people and more’* and *‘% usual residents living in communal setting’* were included as people with close social contact are susceptible to a higher risk of infection.

*Socio-economic variables:*

- Occupation: since working from home and in different occupations and industries have varying risk of exposure to the virus (Beatty & Fothergill, 2021), *‘% of population working in the health sector’* and *‘% of population working from home’* at the MSOA level and *‘% of population in different occupations’* at the LAD level were included.
- Deprivation: after examining *the Index of Multiple Deprivation’s* (IMD) and its domain scores and ranks, only the ‘IMD rank’ was included in analysis to avoid multicollinearity by checking their VIF values and correlation coefficients.

*Built environment variables:*

- Density: since close human contact is associated with the risk of COVID-19 transmission, both area and population densities were calculated, however, only *‘% of built-up area’* at the MSOA level was included as the two measures are highly correlated.
- Road network: it was found highly correlated with density and thus excluded from the analysis.

*Locational variables:*

- Spatial context: the eight supergroups of the ONS 2011 area classification of local authorities^[[2]](#footnote-2)^ were used to measure different spatial contexts. After checking multi-collinearity issues, only six supergroups: *Affluent England; Business, Education & Heritage Centres; Countryside Living; London Cosmopolitan; Services & Industrial Legacy; Urban Settlements* were included as dummy variables.
- Regional effect: the 9 English regions were included as dummy variables to establish wider regional context, except London and East Midlands due to multicollinearity.

**Supplementary Table - Variables and Data Sources**

| **Variables** | **Spatial unit** | **Data source** |
| --- | --- | --- |
| % Population aged 70 and over | MSOA | Mid-2019 Population Estimates, ONS |
| % Population aged 16 to 25 | MSOA | ditto |
| % Households with 2 people | MSOA | 2011 Population Census |
| % Households with 4 people and more | MSOA | ditto |
| % Usual residents living in a communal establishment | MSOA | ditto |
| % Population who are Black | MSOA | ditto |
| % Population who are Indian | MSOA | ditto |
| % Population who are Pakistani | MSOA | ditto |
| % Population who are Bangladeshi | MSOA | ditto |
| % Population who are Chinese | MSOA | ditto |
| % Population who are other Asian | MSOA | ditto |
| Index of Multiple Deprivation (IMD) score/ranking | MSOA | IMD2019 |
| Income deprivation score/ranking | MSOA | English indices of deprivation 2019 |
| Employment deprivation score/ranking | MSOA | ditto |
| Education, skills & training deprivation score/ranking | MSOA | ditto |
| Health deprivation & disability score/ranking | MSOA | ditto |
| Crime score/ranking | MSOA | ditto |
| Barriers to housing & services score/ranking | MSOA | ditto |
| Living environment & deprivation score/ranking | MSOA | ditto |
| % Population working from home | MSOA | 2011 Population Census |
| % Population who work in the health sector | MSOA | Business Register and Employment Survey 2019 |
| % People in employment who are managers, directors, and senior officials | LAD | Annual Population Survey 2020, ONS |
| % People in employment who are in professional jobs | LAD | ditto |
| % People in employment who are in associate professional & technical jobs | LAD | ditto |
| % People in employment who are in administrative and secretarial jobs | LAD | ditto |
| % People in employment who are in skilled trades jobs | LAD | ditto |
| % People in employment who are in caring, leisure and other service jobs | LAD | ditto |
| % People in employment who are in sales and customer service jobs | LAD | ditto |
| % People in employment who are process, plant and machine operatives | LAD | ditto |
| % People in employment who are in elementary jobs | LAD | ditto |
| % Built-up area | MSOA | 2011 Population Census |
| Population density | MSOA | Mid-2019 Population Estimates, ONS |
| Road density | MSOA | OS Open Roads |
| Affluent England | LAD | ONS 2011 area classifications |
| Business, Education & Heritage Centres | LAD | ditto |
| Countryside Living | LAD | ditto |
| Ethnically Diverse Metropolitan Living | LAD | ditto |
| London Cosmopolitan | LAD | ditto |
| Services & Industry Legacy | LAD | ditto |
| Town & Country Living | LAD | ditto |
| Urban Settlements | LAD | ditto |
| East Midlands | LAD | English Boundary datasets, UK Data Service |
| East of England | LAD | ditto |
| London | LAD | ditto |
| North East | LAD | ditto |
| North West | LAD | ditto |
| South East | LAD | ditto |
| South West | LAD | ditto |
| West Midlands | LAD | ditto |
| Yorkshire & The Humber | LAD | ditto |

**Spatial Modelling approaches**

Our test and explanatory variables are at the MSOA and LAD level. We firstly fit a two-level MLM with fixed predictors.


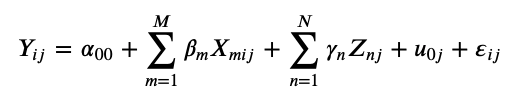


(a)

where *Y_ij_* is the COVID-19 infection rate; *X_mij_* denotes Level 1 variables for MSOA*i* in Level 2 unit of LAD*j*; *Z_nj_* denotes Level 2 variables for LAD*j*; *β_m_* and *γ_n_* represent corresponding coefficients of Levels 1 and 2 variables respectively; *α_00_* is the intercept; *ε_ij_* is the MSOA-level error term; and *u_oj_* represents the LAD-level random intercept.

The application of the MLM to hierarchical geographical data could account for vertical group dependence between the MSOA and the LAD levels but did not capture spatial autocorrelation at the horizontal level as the method assumes spatial independence between different MSOAs. To capture the horizontal spatial dependency, the ESFSM was performed by adopting Moran’s eigenvector spatial filtering technique. The ESFSM is specified as:


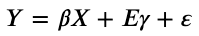


(b)

where *X r*efers to the *i-by-j* data matrix, with *i* number of observations and *j* number of independent variables; *β* denotes the *j-by-1* parameter vector associated with the independent variables; *Eγ* captures spatial dependence defined by the weighted sum of the Moran eigenvectors; and *ε* represents the *n-by-1* spatially independent error vector. Moran eigenvectors are extracted from a doubly-centred spatial proximity matrix. A classic ESFSM considers γ as fixed and *E* as eigenvectors chosen by a stepwise method. However, when the data are with a grouped structure, a single-level ESFSM may induce misleading results with the assumption of statistical independence between different groups.

To capture both vertical and horizontal spatial dependency effects of COVID-19 infection rates, the spatial filtering process was introduced to a multi-level setting. By extending equations (a) and (b), the SFMLM is specified as:


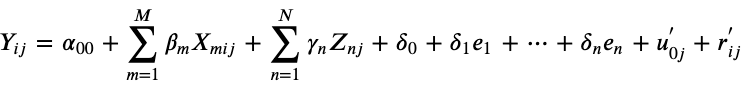


(c)

in which the spatial filtering component *δ_0_ + δ_1_e_1_ + ^…^ + δ_n_e_n_* could account for the spatial effect defined by the Moran eigenvectors; and the component *u'_0j_ + r'_ij_* is the white noise that is independent. The eigenvector filtering process is applied to the MSOA level of the model in this study. The eigenvectors that embody inconsequential levels of spatial dependence were first removed. Significant eigenvectors were then chosen with a stepwise selection method which was performed by maximizing model accuracy and minimising residual spatial dependence.

**Spatial Model Fitness**

Before running different models, *two-level null models for TP1 and TP2* were tested to establish whether the variances of the infection rate were attributed to differences at the LAD level. The estimation results for *TP1* show that the between-LAD variance is 21,746 and the between-MSOA variance is 42,120 (see Table 1), which suggest that 66% of the variance of the infection rate in *TP1* is attributed to the MSOA level (L1) and 34% to the LAD level (L2). For *TP2*, the between-MSOA variation is 707,455 whereas the between-LAD variance is 1,740,129, which means that 71% variation of the infection rate in *TP2* is attributed to the LAD level, with only 29% attributed to the MSOA level (see Table 2). This shows that the spatial effect of the large LAD context has become more significant than the neighbourhood context in explaining infection rates as the pandemic advanced to the second wave.

Following the null models, the *two-level* MLM approach was applied to examine the effects of different test variables in *TP1* and *TP2* respectively. When compared with the null models, both L1 and L2 variances decrease in the MLM 1 and MLM 2. This means that the test variables at the MSOA and LAD levels are explicitly related to the COVID-19 infection rate in *TP1* and that in *TP2*. By addressing the spatial autocorrelation issue of the MSOA, ESFSM was introduced (for data available at the LAD level, the same value was given to the MSOAs nested within it). The resulted Moran’s I values of the estimated spatial filtering processes for *TP1* and *TP2* are 0.157 and 0.359 respectively (see Table 1 and Table 2), which confirms the need to apply the eigenvector spatial filtering process to capture the spatial dependency effect in models for *TP1* and *TP2*. This logically led to the extension of the model by integrating both spatial dependency and multilevel effects by fitting the SFMLM.

The comparison of the three models in Table 1 shows that the explanatory power of some test variables varies. This is true for the three models in Table 2. For instance, of the five occupation variables found significant in the single-level ESFSM 2 only two remained significant in the MLM 2 and SFMLM 2. A comparison of AIC values of the three models for *TP1* suggests that the test variables in SFMLM 1, together with the multi-level and spatial dependency effects, explain 51% of variance in the COVID-19 infection rate in *TP1* and has the highest adjusted R^2^ and lowest AIC values. The SFMLM 1, therefore, stands out as the best-fit model for *TP1*. Similarly, the SFMLM 2 has the best model fitness among the three models examining the infection rate in *TP2* (see Table 2).

The changing Moran’s I values of the estimated spatial process over the two waves suggest that the spatial dependency effect captured by the SFMLM for *TP1* (0.056) is much smaller than that in *TP2* (0.357). The lower R^2^ value for *TP1* (51.1%) further illustrates that there was limited local spatial dependency captured by the Moran eigenvectors than that in *TP2* (82.9%).

1. <https://www.gov.uk/government/publications/full-guidance-on-staying-at-home-and-away-from-others/full-guidance-on-staying-at-home-and-away-from-others> [↑](#footnote-ref-1)
2. <https://www.ons.gov.uk/methodology/geography/geographicalproducts/areaclassifications/2011areaclassifications/maps> [↑](#footnote-ref-2)
